# The Epigenetic Role of Nutrition Among Children and Adolescents: A Systematic Literature Review

**DOI:** 10.3390/children12020143

**Published:** 2025-01-27

**Authors:** Maria Gkiouleka, Maria Karalexi, Theodoros N. Sergentanis, Dimitrios Nouvakis, Stella Proikaki, Eleni Kornarou, Tonia Vassilakou

**Affiliations:** 1 MSc in Public Health, Department of Public Health Policy, School of Public Health, University of West Attica, 196 Alexandras Avenue, 11521 Athens, Greece; maria.gkiouleka@gstt.nhs.uk (M.G.); tsergentanis@uniwa.gr (T.N.S.); stellapro21@gmail.com (S.P.); ekornarou@uniwa.gr (E.K.); 2 Department of Hygiene and Epidemiology, University of Ioannina School of Medicine, 45110 Ioannina, Greece; marykaralexi@windowslive.com; 3 Central Middlesex Hospital, London North West University Healthcare NHS Trust, Harrow HA1 3UJ, UK; dimitrios.nouvakis@nhs.net

**Keywords:** nutrition, physical activity, child, adolescent, epigenetic

## Abstract

Background/Objectives: Recent research has focused on the study of the epigenetic role of nutrition as a tool which is expected to introduce new perspectives in the field of disease prevention and management. Although maternal nutrition is one of the best-studied mechanisms of epigenetic modifications of the fetus/newborn, less is known on the impact of childhood/adolescent nutrition on the regulation of epigenetic mecha-nisms after the first year of life. The aim of the present study was the assessment of the epigenetic role of nutrition in the health and development of children and adolescents. Methods: A systematic review was performed according to the Preferred Reported Items for Systematic Reviews and Meta-analyses guidelines in five databases (PubMed, Cochrane, Science Direct, Scopus, and Google Scholar) up to 31 October 2024, which yielded 17 eligible studies. The Newcastle–Ottawa Scale and the Cochrane Collabora-tion Risk of Bias-2 tool were used for the evaluation of risk of bias in observational studies and randomized trials, respectively. Results: Three studies investigated the epi-genetic modifications due to lifestyle interventions combining changes both in diet and physical activity; the remaining 14 studies examined the role of dietary nutrients in the regulation of epigenetic mechanisms in various health conditions, such as Angelman’s syndrome, parenteral nutrition in Intensive Care Units, attention deficit hyperactivity disorder, risk of cardiovascular diseases, asthma or food sensitization, obesity, insulin resistance, and type 2 diabetes or evaluated epigenetic markers as new tools for the comprehension and prediction of the participants’ response to nutritional interven-tions. Conclusions: The important impact of diet on the regulation of epigenetic mech-anisms and the expression of various genes and gene pathways could be utilized for personalized nutritional interventions in various pediatric health conditions.

## 1. Introduction

Over the past 176 years, since the German philosopher Ludwig Feuerbach stated that “man is what he eats” [1], it has been confirmed that nutrition plays a crucial role in the quality of health in humans. However, on the one hand, large population groups suffer from undernutrition and micronutrient deficiencies (hidden hunger), while Western populations with increased energy intake, unbalanced dietary habits, and reduced physical activity are at high risk of serious non-communicable diseases (NCDs), such as diabetes and obesity [2,3,4]. More specifically, concerning children and adolescents in 2022, globally, 149 million children under the age of five years were stunted, 45 million were wasted, whereas 37 million were overweight or obese. Approximately 50% of children under five years of age’s deaths are attributable to undernutrition. These deaths usually occur in low- and middle-income countries [5]. Furthermore, at least 50% of children under five years old suffer from vitamin and mineral deficiencies globally [6,7]. These conditions often present simultaneously and are interconnected and long-lasting. A healthy diet during the early stages of life is associated with adequate energy and nutrient intake and healthy weight and is pivotal for the physical, cognitive, and mental development of children and adolescents and for their long-term health. Current scientific research related to nutrition has focused on the role of specific dietary components in human health, such as bioactive peptides and proteins, as well as secondary metabolites, which are not only intended to meet energy needs but are also characterized by a wide range of immunomodulatory, antioxidant, osteoprotective, antilipidemic, antithrombotic, and antimicrobial properties [8,9]. In the meantime, there is increased interest in the development of methods for monitoring specific biomarkers in order to design appropriate and more effective dietary interventions [9,10,11].

Epigenetics is a branch of biology that focuses on the causal interplays between genes and their biological products and it constitutes an additional stage in the process of shaping the genome and guiding the transition from the genotype to the phenotype of the individual [12]. Epigenetic mechanisms trigger modifications in the expression of genes by either activating or silencing them, without however causing changes in the deoxyribonucleic acid (DNA) sequence [13]. The main epigenetic mechanisms that shape the epigenetic state of the genome include DNA methylation, histone modification (acetylation, methylation, phosphorylation) and non-coding ribonucleic acids (e.g., miRNAs) [12]. Although epigenetic information is inherited, it is reversible and may be affected by various environmental stimuli (epigenetic plasticity) [14,15]. Environmental epigenetics refers to the way in which the individual’s exposure to environmental factors affects and influences epigenetic mechanisms [16,17,18]. These factors include behaviors, such as diet and physical exercise, as well as exposure to environmental pollutants [19,20]. In other words, lifestyle shapes who we are, changing the epigenome and as a result overall health. Of note is that both the diet of women during pregnancy and the environmental stimuli during the prenatal and early postnatal period of development of the fetus and the newborn can cause permanent changes, which in turn create a predisposition to the onset of diseases in adult life [21]. Maternal nutrition is one of the best-studied mechanisms of epigenetic modifications of the fetus and newborn [22]. However, less is known about the role of childhood and adolescent nutrition in epigenetic regulation after the first year of life. The knowledge of these epigenetic mechanisms may lead to the implementation of targeted personalized nutritional interventions, aiming to prevent or more effectively manage chronic inflammatory and metabolic diseases. Moreover, the tools of epigenetics can be used as prognostic, diagnostic and therapeutic indicators providing new perspectives to the field of prevention [23,24].

Acknowledging these gaps, the aim of the present study is to explore the effect of childhood and adolescent nutrition on inflammatory or metabolic pathways through epigenetic modifications which are associated with various health conditions in later life.

## 2. Materials and Methods

### 2.1. Search Strategy

The systematic literature search was based on a predefined protocol as part of a Master of Science Diploma Thesis and was performed according to the Preferred Re-porting Items for Systematic Reviews and Meta-analyses (PRISMA) guidelines [25]; the PRISMA checklist for the manuscript and abstract are presented as Supplementary Tables S1 and S2, respectively. The systematic review was registered at Open Science Framework (OSF).

We searched five databases, more specifically PubMed, Cochrane, Science Direct, Scopus, and Google Scholar from inception up to 31 October 2024. The terms used for the literature search for the PubMed database are presented in Table 1; the same com-binations of search terms were adopted in the four other databases. Moreover, the PI-COS algorithm that was applied is shown in Table 2.

### 2.2. Inclusion and Exclusion Criteria

Eligible were original studies examining the effect of healthy and unhealthy nutritional habits on mechanisms of epigenetic regulation and modification among children and adolescents aged 1–19 years old, in accordance with the World Health Organization classification for children and adolescents [26]. Only studies in English examining children 1–10 years old or adolescents 10–19 years old were included. The interventions included the dietary habits of minors (fruits, vegetables, fish, meat, dairy, cereals, plant fiber, carbohydrates, fatty acids, sugars) comparing the low and high intake of food components, as well as the healthy and unhealthy habits of these age groups. Study outcomes were all epigenetic mechanisms involved in the process of gene regulation, i.e., DNA methylation, the presence of non-coding RNAs (miRNAs and others) and all histone modifications (methylation, phosphorylation, acetylation).

Studies examining newborns, infants or adults, as well as transgenerational studies and studies not assessing the association of nutrition with epigenetic modifications and their impact on childhood and adolescent health, were excluded.

All article abstracts and full texts were screened by two authors (M.G. and M.K.), working in a blinded fashion. Studies which did not comply with the inclusion criteria were removed. Any controversies were resolved with consensus in a meeting, in which the abstracts and texts were thoroughly reviewed.

### 2.3. Data Extraction and Studies’ Quality Assessment

The data extraction was performed using a predefined Excel sheet including information on: name of first author, date of publication, name of journal, study design, study population, sample size, mean age, age range, type of exposure (type and quantity of food intake), tool of exposure assessment and main outcomes.

The extraction database also included information on the quality of all eligible studies. Specifically, the Newcastle–Ottawa Scale (NOS) was used for the observational studies (cross-sectional, case-control, cohort) [27], while the Risk of Bias-2 tool (RoB 2: a revised tool for assessing risk of bias in randomized trials) was used for randomized clinical trials [28]. In the Newcastle–Ottawa scale, the risk of bias for each study was assessed in three main categories, namely sample eligibility, subgroup comparability, and outcome. The RoB 2 tool for assessing the risk of bias in randomized trials is structured into a fixed set of domains of bias, focusing on different aspects of trial design, conduct, and reporting. Within each domain, a series of questions (“signalling questions”) aim to elicit information about features of the trial that are relevant to risk of bias. Specifically, users of RoB 2 answer one or more of these signalling questions. These answers lead to judgments of “low risk of bias”, “some concerns”, or “high risk of bias” [28].

## 3. Results

### 3.1. Description of Eligible Studies

**Figure 1:**
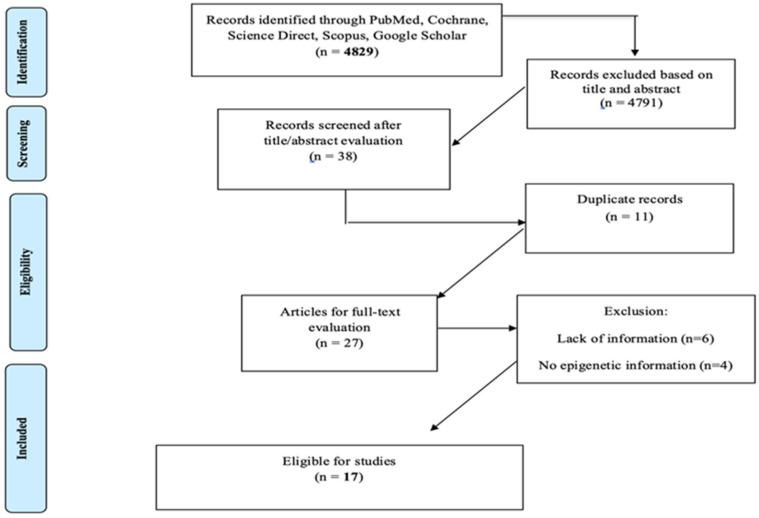
PRISMA flowchart of the systematic review process.

Figure 1 shows the successive steps of the systematic review process. The algorithm search initially yielded 4829 studies, 4791 of which were excluded based on title and abstract screening. Following the exclusion of duplicate publications (n = 11), 27 articles were available for full-text evaluation. After the additional exclusion of 10 articles due to specific reasons, 17 studies were finally eligible to be included in the present systematic review [29,30,31,32,33,34,35,36,37,38,39,40,41,42,43,44,45] (Figure 1).

A total of 17 research studies were examined. Nine studies were observational (five cross-sectional, two cohort studies and two case-control studies) and eight were intervention studies (six randomized and two non-randomized clinical trials [RCTs]). The descriptive characteristics of the eligible studies are presented in Table 3. The qual-ity assessment of the eligible studies is presented in Tables S3 and S4. Variations were found regarding the quality of the included studies. Specifically, among the five cross-sectional studies, one was considered of medium quality (score 6/10), while the re-maining cross-sectional studies were assessed as being of high quality (score > 7/10) according to the NOS scale (Table S3). Both the case-control and cohort studies were considered of high quality (score > 7/9). Among the six RCTs, one was found to be at high risk of bias, for two there were some concerns and for three the risk of bias was low (Table S4). The quality of the two non-RCTs was assessed according to the NOS scale for cohort studies and they were both considered to be of high quality with re-spect to risk of systematic bias (score > 7/9; Table S3).

### 3.2. The Epigenetic Role of Nutrition

Both the two case-control studies refer to Pediatric Early vs. Late Parenteral Nu-trition in Intensive Care Unit (PEPaNIC) RCTs; they compared children admitted in Pediatric Intensive Care Units (PICUs) under early or late parenteral nutrition versus healthy, demographically matched children [29,30]. The first study reported that 159 CpG [cytosine (C) next to guanine (G) connected by a phosphodiester bond (p)] sites in PICU patients followed methylation patterns which differed from those in healthy children—mean effect sizes ranging from 2.6% (standard deviation [SD]: 2.5) to 21.6% (p < 0.02) [29]. Early parenteral nutrition, especially the amount of amino acids, further influenced the observed differences in methylation of 37 (23%) out of the 159 CpG sites (p = 0.0001 to 0.050). In the second study, the methylation pattern in patients was dif-ferent from that in healthy children for 64.6% of the 147 CpG sites on day 3, 72.8% on day 5, and 90.5% on day 7 [30]. In the same context, three cross-sectional studies re-vealed that the methylation levels of specific islands, island shores, and sites were sig-nificantly associated with visceral adiposity and inflammation [31,32,33]. Another cross-sectional study found no DNA methylation differences between infants sensitized and not sensitized to peanuts, eggs, or cow’s milk [34], whereas a recent cross-sectional study reported significant correlations between the nutrient intake of children and the methylation pattern of genes related to obesity (nuclear respiratory factor 1 [NRF-1], fat mass and obesity-associated [FTO], leptin receptor [LEPR] genes) [35]. Among the cohort studies, the first study by Moleres et al. revealed that a calculated methylation score was significantly associated with body weight, body mass index [BMI], standard deviation score (SDS) changes, and body fat mass loss after the intervention [36], whereas according to the second study the detrimental effect of early parenteral nutri-tion on internalizing, externalizing, and overall emotional/behavioral problems was statistically explained by adverse alterations in the methylation of 37 CpG-sites [37].

Among the eight intervention studies, a RCT by Stevens et al. examined the im-pact of a formulation consisting of vitamins, minerals, amino acids, and antioxidants on DNA methylation patterns in children with attention deficit hyperactivity disorder (ADHD; n = 36, age 7–12 years) [38]. Although methylation levels increased at 84% of the most significantly differentially methylated CpG sites, none remained significant after adjusting for genome-wide testing. The olfactory pathway affects the perception of tastes, smells, and food, but its relationship to micronutrient supplementation and DNA methylation is not known. Thus, the general trend toward hypermethylation ob-served in the intervention group suggested that the impact of supplementation on DNA methylation levels corresponded primarily to changes in the expression of small gene loci rather than specific genes, making it highly unlikely that methylation of cyto-sine molecules plays a direct role in the beneficial effect of the supplement in children with ADHD. Two intervention studies comprising children with Angelman syndrome used two dietary supplements, namely betaine and folate, which are donors of methyl groups, attempting to increase the total DNA methylation levels and thereby induce activation of the paternally inherited ubiquitin-protein ligase E3A (UBE3A) gene, which is suppressed in children with Angelman syndrome [39,40]. Both studies con-cluded that no statistically significant changes were observed regarding the develop-mental performance of children who received supplementation. Furthermore, no un-expected changes in biochemical parameters and no alterations in site-specific DNA methylation were reported in participants’ samples obtained pre- and post-treatment. The only significant association was a small, reported reduction in the levels of hyper-active behavior of the children who received the dietary intervention with betaine and folic acid for one year in contrast to those who received a placebo (p = 0.02) [39,40]. In the same context, McMorrow et al. conducted a double-blinded RCT, which examined the impact of an anti-inflammatory nutrition supplement (AINS) comprising long chain n-3 polyunsaturated fatty acid (PUFA), vitamin C, α-tocopherol, green tea ex-tract, and lycopene, which was consumed for 8 weeks, on parameters of the metabolic syndrome in overweight and obese adolescents [41]. This study reported that HMW adiponectin levels were sustained following the AINS, whereas they decreased with the placebo intervention. Additionally, HOMA-IR levels decreased for 40% of the par-ticipants after the AINS [41].

Yadav et al. [42] conducted a RCT to examine the impact of dietary vitamin B12 and/or folate supplementation on genome-wide DNA methylation. While no signifi-cant differences were noticed before supplementation in DNA methylation levels be-tween the participants, significant methylation changes were observed after B12 sup-plementation (589 differentially methylated CpG dinucleotides and 2892 sites), as well as after B12 supplementation with folate (169 differentially methylated CpG dinucleo-tides and 3241 sites). In the folate-supplemented subgroup, 19 differentially methylat-ed CpG sequences were identified, a number almost comparable to that seen in the placebo group (12 differentially methylated CpGs), indicating that these changes may be due to variations over time and not to the dietary intervention. The authors con-cluded that dietary vitamin B12 supplementation (alone or combined with folic acid) may be able to regulate several metabolically important genes linked to Type 2 diabetes (T2D), not only through the differential methylation of the fat mass and obesity-associated (FTO) and transcription factor 7-like 2 (TCF7L2) genes, but also through hypermethylation of a genetic region near the microRNA 21 promoter, providing a novel epigenetic interpretation for the correlation between disturbed carbon 1 (C1) metabolism and the risk of obesity, insulin resistance, and T2D [42]. Likewise, Martins et al. showed that grape juice consumption significantly decreased lipid peroxidation (p = 0.04) and DNA damage (p = 0.01) among male adolescent volleyball athletes after their match [43]. However, levels of interferon (IFN-γ), interleukin 4 (IL-4), N-acetyl-cystein-activated creatine kinase (CK-NAC), and histone H4 acetylation after the match remained unchanged after the grape juice consumption. Lower limb power in-creased after acute exercise in both the placebo and grape juice phases (p < 0.001). Ad-ditionally, a non-RCT examined the effect of lifestyle changes, including personalized Mediterranean diet (MedDiet) in combination with a physical activity program for one year in prepubertal, metabolically healthy individuals [44]. The DNA methylation of all studied genes was significantly altered in the sample after 12 months of lifestyle changes. Correlations were observed between DNA methylation and BMI, homeostatic model assessment of insulin resistance, MUFA and PUFA, moderate-to-vigorous phys-ical activity, fat mass, and adherence to the Mediterranean diet [44]. Lastly, a recent RCT provided evidence that miR-221-3p expression contributes to various obesity-related pathways, targeting specific potential genes linked to childhood obesity, ac-cording to the DisGeNet database [45].

## 4. Discussion

The present systematic review aimed to summarize and synthesize the existing knowledge on the impact of children’s and adolescents’ nutrition on the regulation of epigenetic modifications. The knowledge of these epigenetic mechanisms can lead to the implementation of personalized nutrition programs with the aim of preventing or providing more effective management of chronic pediatric conditions.

### 4.1. Interpretation of Findings and Epigenetic Pathways

Over the recent decades, the term “personalized nutrition” has gained accumulative interest in the fields of Proteomics, Metabolomics, and Nutritional Genomics and is defined as the nutritional approach guided by the genome of each person in order to prevent obesity or treat diseases [46,47]. The C1 metabolic cycle is a very attractive pathway through which environmental influences, such as diet, could directly modify the methylation profile of individuals [48]. The S-Adenosyl-L-methionine (SAM-e) molecule, which results from this metabolic cycle, is the most important donor of methyl groups to the DNA methyltransferases (DNMTs) enzymes, which in turn catalyze the conversion reaction of cytosine to 5-methylcytosine (5mC) [49]. Any disturbances in the bioavailability of SAM-e can result in alterations to the body’s methylome, which offers a fairly clear explanation of the mechanism by which diet alters methylation levels, affect gene regulation and gene expression [49].

Among the studies included in the present systematic review, two intervention studies concerned Angelman syndrome [39,40]. In both these studies, the dietary supplementation with betaine and folic acid or with betaine, metafolin, creatine, and vitamin B12, appeared safe, but ineffective in reducing the severity of Angelman syndrome symptoms. However, the improvement trend observed in some of the developmental parameters evaluated could be the trigger for conducting studies using more and stronger methyl group donors, which would promote overall DNA methylation levels, ultimately ameliorating the clinical phenotype of the syndrome [50,51]. Similarly, an intervention with a dietary micronutrient supplementation was found to have no significant biological effect on DNA methylation over 10 weeks, possibly attributed to the short period of exposure to the dietary supplement [38]. Noteworthy were the findings of studies examining DNA methylation as a new tool for understanding the effect of diet on obesity and metabolic syndrome phenotypes. The study by Moleres et al. was the first to calculate an overall predisposing epigenetic score for obesity [36]. The results of this study showed that the epigenetic profile of individuals, which is modelled according to the cumulative epigenetic score of differentially methylated CpG before the implementation of the dietary intervention among participants with low or high response, can predict the reduction of BMI and the improvement of other anthropometric measurements at the end of the program [36]. Therefore, this epigenetic score could be used as a prognostic tool to predict the outcome of a multidisciplinary program including changes in lifestyle and dietary habits for weight loss. Of particular interest were also the findings of Yadav et al. and McMorrow et al., according to which specific dietary interventions could affect the occurrence of T2D and obesity [41,42]. Moreover, the study by McMorrow et al. suggested that an anti-inflammatory dietary intervention (AINS) may affect the methylation level of genes involved in the pathophysiology of obesity and highlighted that the implementation of more stratified or individualized nutritional approaches could enhance the effectiveness of nutritional interventions aimed to protect against T2D [41]. Future studies could further assess whether these changes in the epigenetic machinery of DNA methylation cause or are the result of the phenotypic response to therapeutic interventions. Likewise, according to Yadav et al., dietary vitamin B12 supplementation (either alone or in combination with folic acid) affected the regulation of several metabolically important genes associated with T2D, not only through differential methylation of the FTO and TCF7L2 genes but also through the hypermethylation of a genetic region near the miRNA 21 promoter, providing a new epigenetic explanation for the association between disturbed C1 metabolism and the risk of obesity, insulin resistance, and T2D [52,53]. Moreover, the study by Ojeda-Rodríguez et al. showed that specific miRNAs are associated with abdominal obesity; specifically, the miR-221-3p participates in several obesity-related pathways in young girls with abdominal obesity and thus, it could be used for the prediction of the individuals’ response to a multidisciplinary intervention aiming at weight loss among young girls [45].

Numerous studies have shown that both the type and quantity of dietary fat greatly affect the metabolic pathways which lead to the development of obesity, metabolic syndrome, T2D, and cardiovascular diseases [54,55,56,57,58]. However, the extent to which the epigenetic mechanism of methylation is involved in this process has not yet been clarified. The study by Voisin et al. found that the quality of dietary fat can influence DNA methylation on a large scale, as gene enrichment analyses identified the presence of significant signalling pathways for the three dietary fatty acid intake ratios studied [PUFA/SFA, MUFA/SFA, (MUFA + PUFA)/SFA] [31]. In the clinical trial by Chen et al., three CpG sequences were found, located in the lysophosphatidylcholine acyltransferase 1 (LPCAT1), and RAS P21 Protein Activator 3 (RASA3) genes, whose methylation levels were significantly correlated with dietary fiber intake [32]. Methylation of two of these three CpG sites was also found to be associated with visceral fat deposition and inflammation. Accordingly, two of the four most significantly differentially methylated CpG dinucleotide sequences appeared to be associated with both dietary fiber consumption and inflammatory mechanism activation and visceral fat deposition, with this finding suggesting that dietary fiber may indirectly affect the epigenetic mechanisms of DNA methylation [32]. Future studies are needed to determine whether epigenetic regulation (via methylation of specific CpG sites) underlies the beneficial effects of dietary fiber on inflammation and body fat deposition. The epigenetic role of nutrition can also be positive in children with asthma, as found by Montrose et al. [33]. The study reported that selenium and other methyl-donor dietary nutrients, such as phosphocholine, betaine, and folate, were found to be positively associated with the measure of asthma quality of life, achieving greater scores on the Pediatric Asthma Quality of Life Questionnaire (PAQLQ); this finding could be useful in recommending new protocols for the management of childhood asthma in the future [33].

### 4.2. Implications and Future Directions for Clinical Practice

To further investigate the epigenetic impact of nutrition on the health of children and adolescents, additional research is deemed necessary. More specifically, future studies could focus on either the use of more and more potent methyl group donors, which will promote overall DNA methylation levels or the longer period of exposure of participants to nutritional interventions. In this context, it may be possible to determine whether the regulation of epigenetic mechanisms (mainly through the methylation of specific CpG sites) is the main causal basis for the favourable effects that various nutritional components have been reported to exhibit on the mechanisms of inflammation, body fat deposition and the occurrence of obesity, type 2 diabetes, and cardiovascular diseases. At the same time, it is important to conduct more clinical trials among children and adolescents, as they are necessary, in order to study the epigenetic role of nutrition during childhood and adolescence, both in terms of histone modifications and non-coding RNAs, as these two mechanisms have not been investigated in depth, compared to the DNA methylation process. This could yield more accurate results on the interaction between the nutritional interventions and the three basic epigenetic mechanisms.

An enhanced knowledge of epigenetic mechanisms may lead to the implementation of targeted personalized nutritional interventions, aiming to prevent or more effectively manage chronic inflammatory diseases, in comparison with more traditional practices. Moreover, the tools of epigenetics can be used as prognostic, diagnostic, and therapeutic indicators providing new perspectives to the field of prevention and clinical practice, leading to improved health outcomes [20,21]. Examples of the potential applications according to the reported findings of the present review include the use of an epigenetic score as a prognostic tool to predict the outcome of multidisciplinary lifestyle and nutritional programs for weight loss and the implementation of personalized dietary interventions (e.g., anti-inflammatory diets, dietary vitamin B12 and/or folic acid supplementation, modification of the quality and amount of dietary fat, changes in dietary fiber intake) that could affect the occurrence of T2D and/or obesity via various epigenetic mechanisms and pathways [59,60,61,62]. Moreover, nutrition can also play a positive epigenetic role in the quality of life among children with asthma.

Lastly, healthy nutrition educational programs that will aim to improve diet, quality of life, and health of children and adolescents, based on the principles of prevention and modification of health-influencing behaviors and disease prevention, are highly recommended [63,64,65]. Such programs can be initiated through interventions in schools which will focus on expanding students’ knowledge regarding nutritional components, healthy dietary patterns, their interaction with the genome and epigenome, but also about the important role of physical activity in the regulation of epigenetic mechanisms and consequently in the expression of various genes and gene pathways with the aim of improving their attitudes and behaviors regarding healthy nutrition and regular physical activity.

### 4.3. Strengths and Limitations

Although this is the first systematic review that examined the impact of dietary components on the formulation and modification of epigenetic mechanisms in children and adolescents, a number of limitations need to be acknowledged. The most important limitation is the relatively small number of primary studies that have been conducted in children and adolescents, since the majority of published literature, which focused on the epigenetic effect of nutrition, includes transgenerational studies, examining the role of maternal nutrition during pregnancy and early postnatal period in the regulation of epigenetic mechanisms of the offspring during the postpartum period. In addition, almost all intervention studies had relatively short duration of interventions, which could have affected their reliability and outcomes, given that the majority of the epigenetic changes occur after long-term exposure to environmental factors, such as diet. Finally, the sample sizes in both observational and intervention studies were relatively small, thus not allowing us to reach robust conclusions.

## 5. Conclusions

The present systematic review based on 17 primary studies revealed promising results regarding the role of epigenetic mechanisms as tools for predicting participants’ response to nutritional interventions. Specifically, the epigenetic profile of individuals may be shaped according to cumulative epigenetic scores based on differentially methylated CpG sequences. Moreover, although the administration of dietary supplements rich in methyl-donor components did not directly improve the phenotype of the targeted diseases, an improvement trend was observed in some of the evaluated parameters. Thus, the magnitude of changes noted in the overall methylation levels in the whole genome could be the trigger for future studies focused either on longer periods of exposure to the dietary interventions or the use of more and stronger donors of methyl groups, which could promote the overall levels of DNA methylation. Lastly, the ultimate goal is to organize health and nutrition educational programs that aim to improve the health of children and adolescents based on the principles of prevention and modification of lifestyle behaviors. These programs can be initiated through interventions in schools with the aim of expanding students’ knowledge about individual nutritional components, healthy dietary patterns, their interaction with the genome and epigenome, but also about the important role of diet in the regulation of epigenetic mechanisms and therefore in the expression of various genes and gene pathways with the aim of improving their attitudes and behaviors regarding nutrition and physical activity.

## Figures and Tables

**Table 1:** Keywords for the PubMed database.

Search String
(children OR adolescents) AND (diet OR dietary OR nutrition OR food OR cereal OR carbohydrates OR fiber OR fruit OR vegetables OR fish OR meat OR sugar OR oil OR dairy) AND (“DNA methylation” OR “epigenetic” OR “MicroRNAs” [Mesh] OR “Histone Code” [Mesh] OR “Epigenome” [Mesh] OR Epigenomics [Mesh])

**Table 2:** PICOS acronym for the systematic review.

Participants	Intervention	Comparison	Outcomes	Study Design
Children and adolescents (1–19 years old)	Dietary habits	Healthy versus unhealthy nutrition	Mechanisms of epigenetic regulations/modifications	Primary studies

**Table 3:** Descriptive characteristics of the eligible studies.

Author, Year of Publication	Study Design	Sample Size	Assessment Tool	Exposure Ascertainment	Study Results
**Case-control studies**					
Güiza et al. (2020) [29]	Case-control; secondary analysis of PEPaNIC trial	Cases: n = 825 children aged 0–17 years at PICU; controls: n =352 demographically matched healthy children	Infinium Human MethylationEPIC BeadChip	Early and late parenteral nutrition	159 CpG sites exhibited differential methylation between PICU patients and healthy children. The mean effect size of methylation differences ranged from 2.6% to 21.6% (p < 0.02. Early parenteral nutrition (PN), mainly the amount of amino acids, contributed to the observed methylation differences of 37 (23%) of the 159 CpG sites (p = 0·0001 to 0·050).
Verlinden et al. (2020) [30]	Case-control; secondary analysis of PEPaNIC trial	Children under early (n = 36) and late (n = 36) parenteral nutrition versus healthy children (n = 42)	Infinium^®^ HumanMethylation EPIC BeadChip	Early and late parenteral nutrition	In patients, methylation patterns differed from those in healthy children at 64.6% of the 147 CpG-sites by day 3, 72.8% by day 5, and 90.5% by day 7. A rapid demethylation was observed from admission to day 3 for 76.2% of the CpG-sites. Additionally, 19.7% of the CpG-sites became hypermethylated from admission to day 3, while only 4.1% of the CpG-sites showed changes beyond day 3.
**Cross-sectional studies**					
Voisin et al. (2015) [31]	Cross-sectional	69 Greek preadolescents (~10 years old)	Illumina Infinium HumanMethylation27 BeadChip and food-frequency questionnaires	Cholesterol intake, percentage of energy intake from fat, MUFA + PUFA to SFA PUFA/SFA, MUFA/SFA (MUFA + PUFA)/SFA	The methylation levels of 2 islands, 11 island shores, and 16 sites showed significant correlation with PUFA/SFA. Additionally, methylation levels of 9 islands, 26 island shores, and 158 sites associated with MUFA/SFA, while of 10 islands, 40 island shores, and 130 sites associated with (MUFA + PUFA)/SFA.
Chen et al. (2018) [32]	Cross-sectional	African American adolescents (n = 284)	Illumina 450K Bead-Chip. Structured 24-h food surveys	Dietary Fiber Intake	The methylation levels of 3 CpG sites (cg15200711, cg19462022, and cg07035602) in LPCAT1 and RASA3 genes were correlated with fiber intake (FDR < 0.05). The methylation levels of cg07035602 and cg19462022 correlated with visceral adiposity and inflammation.
Montrose et al. (2017) [33]	Cross-sectional	A total of 32 children with asthma residing in western Montana were recruited for the ARTIS study	Food frequency questionnaire. Pyrosequencing assays for methylation sites	Nutrition	Selenium and various other methyl- donating nutrients were positively linked to the quality of life of children with asthma. The intake of methyl-donating nutrients, such as folate, was positively correlated with LINE-1 methylation and negatively correlated with IFNγ CpG-186. Additionally, higher LINE-1 methylation was linked to higher dPFV.
Kolsun et al. (2023) [34]	Cross-sectional	n = 144 children with early asthma symptoms recruited to the CHILD REEGLE subset cohort	Food-frequency questionnaire, skin prick testing, Illumina HumanMethylation450k array for DNA methylation	Peanuts, eggs, or cow’s milk sensitization by the age of 12 months	There were no differences in DNA methylation between infants sensitized and not sensitized to peanuts, eggs, or cow’s milk. However, borderline significant sites with high effect sizes were enriched for methylation quantitative trait loci, suggesting that genetic factors may influence DNA methylation at these sites.
Patel et al. (2023) [35]	Cross-sectional	Children aged 6–10 years from counties Lee and Macon (n = 113)	Food-frequency questionnaire, body weights and heights measurements, MethyLight RT-PCR reaction and multiplex PCR	Dietary intake	There were significant correlations between children’s nutrient intake and the methylation of the NRF1, FTO, and LEPR genes.
**Cohort studies**					
Moleres et al. (2013) [36]	Cohort	Overweight or obese adolescents (n = 36)	Human Methylation 27 Bead Chip and MALDI-TOF mass spectrometry. Food frequency questionnaires	A 10-week multidisciplinary program aiming at weight loss	Five regions located in or near AQP9, DUSP22, HIPK3, TNNT1, and TNNI3 genes showed differences in methylation levels between high and low responders to the weight loss intervention. A calculated methylation score significantly correlated with weight, BMI-SDS, and body fat mass loss by the end of the intervention.
Jacobs et al. (2021) [37]	Cohort	Children receiving late parenteral nutrition (n = 211) vs. those receiving early parenteral nutrition (n = 192)	Combination of DNA methylation data with behavioral data collected 4 years post-PICU admission	Early and late parenteral nutrition	The detrimental effect of early parenteral nutrition on internalizing, externalizing, and overall emotional/behavioral problems was statistically explained by adverse alterations in the methylation of 37 CpG-sites.
**Intervention studies**					
Stevens et al. (2018) [38]	RCT	Children with ADHD (n = 36)	Infinium Methylation EPIC 850 K	A formulation that included a mixture of vitamins, minerals, amino acids, and antioxidants	Methylation levels increased at 84% of the most significantly differentially methylated CpG sites; however, none remained significant after adjusting for genome-wide testing.
Bird et al. (2011) [39]	Non-RCT	Children with Angelman syndrome (n = 65)	Neuropsychological assessments, biochemical analyses, and DNA methylation evaluations with Human Methylation 27 Bead Chip array	Dietary supplementation including betaine, metafolin, creatine, and vitamin B12 for 1 year	No statistically significant changes were observed in the developmental performance of children who received dietary supplementation. Furthermore, no unexpected changes of biochemical parameters and no alterations in site-specific DNA methylation were found between samples of participants obtained pre- and post- treatment.
Peters et al. (2010) [40]	RCT	Children with Angelman syndrome (n = 48)	Quattro Micro tandem mass spectrometer; Developmental assessments, biochemical analyses of blood and urine, and electroencephalographic examinations	A dietary supplementation with betaine and folic acid for 1 year	There were no statistically significant differences between treated and untreated children; however, a small subset of patients exhibited some positive trends.
McMorrow et al. (2018) [41]	Double-blinded RCT	Overweight and obese adolescents (n = 70)	HOMA-IR, adiponectin levels, inflammatory indices, and DNA methylation (Infinium HumanMethylation450 BeadChip assay)	AINS: supplementation with long-chain n-3 PUFA, vitamin C, α-tocopherol, green tea extract, and lycopene taken for 8 weeks	High-Molecular-Weight (HMW) adiponectin levels were sustained following the AINS, whereas they decreased with the placebo intervention. Additionally, HOMA-IR levels decreased for 40% of the participants following the AINS.
Yadav et al. (2017) [42]	RCT	Children participated in the PMNS trial (n = 48)	Infinium HumanMethylation450 BeadChip	Supplementation with B12 and/or folic acid	Post-supplementation, significant methylation changes were observed in the B12 group (589 differentially methylated CpG sites and 2892 regions) and the B12 plus folic acid group (169 differentially methylated CpG sites and 3241 regions). Methylation influences miR21 expression, and FTO, TCF7L2, CREBBP/CBP, and SIRT1 are direct targets of miR21-3p. Significant methylation changes post-supplementation in B12 (589 differentially methylated CpG and 2892 regions) and B12 plus folic acid (169 differentially methylated CpG and 3241 regions) groups. Methylation affected miR21 expression, while FTO, TCF7L2, CREBBP/CBP and SIRT1 are direct targets of miR21-3p.
Martins et al. (2020) [43]	Double-blinded RCT	12 male adolescent volleyball athletes (aged 16 ± 0.6 years old)	Indicators of systemic redox status, systemic concentrations of interferon (IFN-γ) and interleukin-4 (IL-4), muscle damage (assessed by N-acetyl-cystein-activated creatine kinase (CK-NAC)), and levels of global histone H4 acetylation were estimated. Additionally, HG and lower limb power tests were performed	Grape juice (400 mL) vs. placebo consumption (400 mL) for 14 days	Grape juice consumption significantly decreased lipid peroxidation (p = 0.04) and DNA damage (p = 0.01) post-match. However, levels of IFN-γ, IL-4, CK-NAC, and histone H4 acetylation after the match remained unchanged with the grape juice consumption. Lower limb power increased after acute exercise in both the placebo and grape juice phases (p < 0.001).
Gallardo-Escribano et al. (2020) [44]	Non-RCT	Prepubertal subjects with metabolically healthy obesity (n = 131)	HOMA-IR assessment, DNA methylation arrays	Personalized MedDiet combined with a physical activity program for 1 year	The DNA methylation of all studied genes was significantly altered in the sample after 12 months of lifestyle changes. Correlations were observed between DNA methylation patterns and BMI, homeostatic model assessment of insulin resistance, MUFA and PUFA, moderate-to-vigorous physical activity, fat mass, and adherence to the Mediterranean diet.
Ojeda-Rodríguez et al. (2022) [45]	RCT	51 Spanish girls (age 7–16 years) in the IGENOI study	HOMA-IR, anthropometric, clinical and biochemical measurements	An 8-week intervention, including nutritional education based on the Mediterranean diet and an increase in physical activity to at least 200 min per week	Six miRNAs were differentially expressed between the low and high responders. After adjusting for Tanner stage, the correlation remained significant only for miR-126-3p and miR-221-3p, with higher expression in the high responder group compared to the low responder group. Following the intervention, miR-221-3p expression reduced in all subjects, with a significant difference in the change within groups. The expression of miR-221-3p was found positively associated with body weight, BMI, and waist circumference, and negatively associated with the quantitative insulin sensitivity check index.

Abbreviations: PEPaNIC: Pediatric Early vs. Late Parenteral Nutrition in Intensive Care Unit; CpG: cytosine (C) next to guanine (G) connected by a phosphodiester bond (p); PICU: Pediatric Intensive Care Unit; SD: standard deviation; MUFA: monounsaturated fatty acid; PUFA: polyunsaturated fatty acid: SFA: saturated fatty acid; LPCAT1: lysophosphatidylcholine acyltransferase 1; RASA3: RAS p21 protein activator 3; FDR: False Discovery Rate; ARTIS: Recombinant t-PA Thrombolys in Ischemic Stroke; LINE-1: Long interspersed nuclear element-1; IFN: interferon; dPFV: diastolic mean peak flow velocity; AQP9: Aquaporin 9; DUSP22: dual specificity phosphatase 22; HIPK3: Homeodomain-interacting protein kinase 3; TNNT1: Slow skeletal muscle troponin T 1; TNNI3: troponin I3, cardiac type; BMI-SDS: body mass index standard deviation score; ADHD: Attention-deficit/hyperactivity disorder; AINS: Anti-inflammatory nutrition supplement; HOMA-IR: Homeostatic model assessment; HMW: High-molecular-weight; IL-4: Interleukin-4; CK-NAC: Creatine Kinase; HG: handgrip strength; DNA: Deoxyribonucleic Acid; MedDiet: Mediterranean diet; RCT: randomized controlled trial; PMNS: Pune Maternal Nutrition Study; IGENOI: Intervention of Grupo de Estudio Navarro de Obesidad Infantil; NRF1: Nuclear respiratory factor 1; FTO: Fat mass and obesity-associated: LEPR: leptin receptor.

## Data Availability

The original contributions presented in the study are included in the article/Supplementary Materials; further enquiries can be directed to the corresponding author.

## Supplementary Materials

The following supporting information can be downloaded at: https://www.mdpi.com/article/10.3390/children12020143/s1, Table S1: PRISMA 2020 Checklist; Table S2: PRISMA 2020 for Abstract s Checklist; Table S3: Quality as-sessment results of case-control, cross-sectional, cohort and non-randomized clinical trials based on the Newcastle–Ottawa Scale; Table S4: Risk of bias of randomized clinical trials (Rob 2).
