# Supplementary material for: The Epigenetic Role of Nutrition Among Children and Adolescents: A Systematic Literature Review
Source: Children (Basel). 2025 Jan 27;12(2):143. doi: 10.3390/children12020143 (PMC11854644; doi:10.3390/children12020143)
Supplement: Supplementary file 1 [file children-12-00143-s001.zip › Supplementary Material/Tables S3 and S4.docx]

**Supplementary material Tables S3 and S4**

**Table S3.** Quality assessment results of case-control, cross-sectional, cohort and non-randomized clinical trials based on the Newcastle-Ottawa Scale

| **Case-control studies** | | | | |
| --- | --- | --- | --- | --- |
|  | **Selection (up to 4)** | **Comparability (up to 2)** | **Outcome (up to 3)** | **Total (up to 9)** |
| Güiza et al., 2020 | 4 | 1 | 2 | 7 |
| Verlinden et al., 2020 | 4 | 1 | 2 | 7 |
| **Cross-sectional studies** |  |  |  |  |
|  | **Selection (up to 5)** | **Comparability (up to 2)** | **Outcome (up to 3)** | **Total (up to 10)** |
| Montrose et al., 2017 | 2 | 1 | 3 | 6 |
| Chen et al., 2018 | 3 | 2 | 3 | 8 |
| Voisin et al., 2015 | 3 | 2 | 3 | 8 |
| Kolsun et al., 2023 | 4 | 2 | 3 | 9 |
| Patel et al., 2023 | 3 | 2 | 3 | 8 |
| **Cohort and non-Randomized Clinical Trials** | | | | |
|  | **Selection (up to 4)** | **Comparability (up to 2)** | **Outcome (up to 3)** | **Total (up to 9)** |
| Moleres et al., 2013 | 3 | 1 | 3 | 7 |
| Jacobs et al., 2021 | 4 | 1 | 3 | 8 |
| Gallardo-Escribano et al., 2020 | 3 | 2 | 3 | 8 |
| Bird et al., 2011 | 4 | 2 | 3 | 9 |

**Table S4.** Risk of bias 2 tool of randomized clinical trials (Rob 2)

| **Domains** | **Stevens et al., 2018** | **Peters et al., 2010** | **McMorrow et al., 2018** | **Yadav et al., 2017** | **Martins et al., 2020** | **Ojeda-Rodríguez et al., 2022** |
| --- | --- | --- | --- | --- | --- | --- |
| **Risk of bias arising from the randomization process** | Low risk | Low risk | Low risk | Low risk | Low risk | Low risk |
| **Risk of bias due to deviations from the intended interventions** | Low risk | Low risk | Low risk | Low risk | Low risk | Low risk |
| **Bias due to missing outcome data** | Low risk | Some concerns | Some concerns | Low risk | Low risk | Low risk |
| **Risk of bias in measurement of the outcome** | Low risk | Low risk | Low risk | High risk | Low risk | Low risk |
| **Risk of bias in selection of the reported result** | Low risk | Some concerns | Low risk | Some concerns | Low risk | Low risk |
| **Overall risk of bias** | Low risk | Some concerns | Some concerns | High risk | Low risk | Low risk |
